# HIV Infection and HIV-Associated Behaviors Among Persons Who Inject Drugs — 20 Cities, United States, 2015

**DOI:** 10.15585/mmwr.mm6701a5

**Published:** 2018-01-12

**Authors:** Janet C. Burnett, Dita Broz, Michael W. Spiller, Cyprian Wejnert, Gabriela Paz-Bailey

**Affiliations:** 1 Division of HIV/AIDS Prevention, National Center for HIV/AIDS, Viral Hepatitis, STD, and TB Prevention, CDC.

In the United States, 9% of human immunodeficiency virus (HIV) infections diagnosed in 2015 were attributed to injection drug use (*1*). In 2015, 79% of diagnoses of HIV infection among persons who inject drugs occurred in urban areas (*2*). To monitor the prevalence of HIV infection and associated behaviors among persons who inject drugs, CDC’s National HIV Behavioral Surveillance (NHBS) conducts interviews and HIV testing in selected metropolitan statistical areas (MSAs) (*3*). The prevalence of HIV infection among persons who inject drugs in 20 MSAs in 2015 was 7%. In a behavioral analysis of HIV-negative persons who inject drugs, an estimated 27% receptively shared syringes and 67% had condomless vaginal sex in the previous 12 months. During the same period, 58% had tested for HIV infection and 52% received syringes from a syringe services program. Given the increased number of persons newly injecting drugs who are at risk for HIV infection because of the recent opioid epidemic (*2*,*4*), these findings underscore the importance of continuing and expanding health services, HIV prevention programs, and community-based strategies, such as those provided by syringe services programs, for this population.

In 2015, NHBS staff members in 20 MSAs* collected cross-sectional behavioral survey data and conducted HIV testing among persons who inject drugs; survey participants were recruited using respondent-driven sampling (RDS),^†^ a peer-referral sampling method (*5*). Eligible participants^§^ completed a standardized questionnaire administered face-to-face by trained interviewers. All participants were offered anonymous HIV testing^¶^; a nonreactive screening test result was considered HIV-negative and a reactive screening test result was considered HIV-positive if confirmed by western blot or indirect immunofluorescence assay. Incentives were offered for completing the interview, HIV testing, and recruitment.** Participants were asked about behaviors in the previous 12 months, including high-risk injection (receptive sharing)^††^ or sexual behaviors,^§§^ testing for HIV and hepatitis C virus (HCV) infection, participation in HIV behavioral interventions,^¶¶^ and receiving any syringes from a syringe services program or all syringes from sterile sources.*** Because knowledge of personal HIV infection status could influence risk behaviors (*6*), behavioral analysis was limited to HIV-negative persons who inject drugs.^†††^ Data from each MSA were analyzed using the RDS Analysis Tool that produces estimates adjusted for differences in peer recruitment patterns and the size of the network of persons who inject drugs and estimated 95% confidence intervals (CIs) (*5*). All comparisons were considered significant if there was no overlap in their adjusted 95% CIs; because of the sampling methodology, RDS analysis is limited to calculating point estimates with CIs and precludes any other statistical testing. A weighted average of MSA-level estimates was calculated using the estimated size of the population of persons who inject drugs in each MSA to describe aggregated prevalence of HIV and percentage of participants engaging in selected behaviors (*7*).^§§§^

In 2015, 13,633 persons were recruited to participate; 2,955 (22%) were ineligible and 330 (3%) were excluded because of incomplete data.^¶¶¶^ Among the 10,348 persons who injected drugs who tested for HIV, 709 (6.9%) tested HIV-positive and 9,639 tested HIV-negative. Adjusted HIV prevalence in the 20 MSAs was estimated to be 7% (Table 1). HIV prevalence was higher**** among blacks (11%) than whites (6%) and among persons in the South U.S. Census region (10%) than in the Midwest (3%) and Northeast (5%) regions. The prevalence of HIV infection was 24% among males who inject drugs who reported male-to-male sex in the previous 12 months.

**TABLE 1:** Estimated prevalence of human immunodeficiency virus (HIV) infection among persons who inject drugs (N = 10,348), by selected characteristics — National HIV Behavioral Surveillance, 20 cities, United States, 2015

Characteristic	Overall*	HIV prevalence*
	% (95% CI)	% (95% CI)
**Overall**	**100 — **	**7(6–8)**
**Sex**		
Men	69(67–71)	6 (5–7)
Women	31(29–33)	9 (7–12)
**Race/Ethnicity**		
Black, non-Hispanic	39 (36–42)	11 (8–15)
Hispanic^†^	21(19–23)	7 (4–9)
White, non-Hispanic	39 (36–41)	6 (5–8)
Other^§^	2 (1–2)	—**^¶^**
**Age group (yrs)**		
18–29	14 (12–16)	2(1–3)
30–39	21(19–23)	5(3–6)
40–49	24(22–26)	11(9–13)
≥50	41(39–44)	9(7–11)
**Education**		
Less than HS diploma	28 (26–30)	8(4–12)
HS diploma	41(39–44)	8 (6–9)
More than HS diploma	31 (29–33)	6 (0–13)
**Health insurance**		
No	18 (17–20)	3(2–4)
Yes	82 (80–83)	8 (6–9)
Poverty level**		
At or below FPL	78 (76–79)	7 (6–9)
Above FPL	22(21–24)	6 (5–8)
**Drug injected most frequently**		
Heroin only	65(63–67)	5(2–8)
Other/Multiple**^††^**	35(33–37)	11(9–13)
**Male-male sex, last 12 months (among males only)**		
No	90(88–93)	5(3–6)
Yes	10(7–12)	24(15–33)
**U.S. Census region** ^§§^		
Northeast	24(24–51)	5(3–7)
South	36(15–42)	10(8–13)
Midwest	11(0–22)	3(1–5)
West	24(10–37)	7(5–9)

**Abbreviations**: CI = confidence interval; FPL = federal poverty level; HS = high school.

* Aggregate estimates are weighted averages of MSA (metropolitan statistical areas) -level percentages. MSA-level percentages were adjusted for differences in recruitment and the size of participant peer networks of persons who inject drugs, then proportionally weighted by the size of the population of persons who inject drugs in each city.

^†^ Persons of Hispanic ethnicity might be of any race or combination of races.

^§^ Includes American Indian/Alaska Natives, Asians, Native Hawaiian or other Pacific Islanders, and persons of multiple races.

^¶^ Insufficient data.

** Poverty level is based on household income and household size.

^††^ Other drugs injected alone or two or more drugs injected with the same frequency.

^§§^ The Northeast region includes the MSAs of Boston, Massachusetts; Nassau-Suffolk, New York; New York, New York; Newark, New Jersey; and Philadelphia, Pennsylvania. South region includes Atlanta, Georgia; Baltimore, Maryland; Dallas, Texas; Houston, Texas; Miami, Florida; New Orleans, Louisiana; and Washington, District of Columbia. Midwest region includes Chicago, Illinois and Detroit, Michigan. West region includes Denver, Colorado; Los Angeles, California; San Diego, California; San Francisco, California; and Seattle, Washington. San Juan, Puerto Rico was not included.

Among the HIV-negative participants, 27% receptively shared syringes, 67% had condomless vaginal sex, 22% had condomless heterosexual anal sex, and 45% had more than one opposite sex partner (Table 2). Receptive syringe sharing was higher among whites (39%) than among Hispanics (24%) and blacks (17%); similar patterns were seen for sharing injection equipment (61%, 45%, and 41%, respectively). Condomless vaginal and anal sex was higher among whites (74% and 25%, respectively) than among blacks (62% and 17%, respectively).

**TABLE 2:** Estimated percentage* of HIV-negative participants who inject drugs (n = 9,639) who engaged in behaviors^†^ associated with HIV infection in the previous 12 months, by selected characteristics — National HIV Behavioral Surveillance, 20 cities, United States, 2015

Characteristics	Receptive syringe sharing,^†^ % (95% CI)	Receptive injection equipment sharing,^†^ % (95% CI)	Had vaginal sex, % (95% CI)	Had condomless vaginal sex, % (95% CI)	Had heterosexual anal sex, % (95% CI)	Had condomless heterosexual anal sex, % (95% CI)	Had condomless heterosexual sex or receptive syringe sharing, % (95% CI)	Had more than one opposite sex partner, % (95% CI)
**Overall**	**27 (25–29)**	**49 (46–51)**	**78 (76–80)**	**67 (65–70)**	**28 (26–30)**	**22 (20–24)**	**72 (70–75)**	**45 (42–47)**
**Sex**								
Men	27 (25–29)	48 (46–51)	77 (74–79)	65 (63–68)	27 (25–29)	20 (19–22)	73 (71–75)	44 (42–47)
Women	28 (24–31)	49 (45–54)	82 (78–86)	73 (68–77)	29 (24–34)	24 (20–29)	77 (72–81)	44 (39–48)
**Race/Ethnicity** ^§^								
Black, non-Hispanic	17 (14–19)	41 (37–45)	75 (72–79)	62 (58–66)	22 (19–24)	17 (14–19)	68 (65–71)	41 (38–45)
Hispanic^¶^	24 (20–27)	45 (41–49)	79 (74–84)	68 (62–73)	33 (28–38)	26 (22–31)	74 (69–79)	43 (38–47)
White, non-Hispanic	39 (35–42)	61 (57–64)	82 (79–85)	74 (71–77)	31 (27–34)	25 (22–28)	81 (78–84)	48 (44–52)
**Age group (yrs)**								
18–29	41 (36–46)	63 (56–69)	89 (85–93)	80 (75–85)	43 (36–49)	33 (28–39)	85 (80–91)	62 (56–68)
30–39	38 (33–42)	58 (53–62)	90 (87–92)	82 (78–85)	37 (31–42)	30 (25–35)	86 (83–89)	53 (48–58)
40–49	25 (22–28)	47 (42–52)	77 (72–81)	69 (65–73)	27 (23–30)	20 (18–23)	76 (72–80)	41 (37–46)
≥50	17 (14–19)	41 (38–45)	68 (65–72)	56 (53–60)	18 (15–20)	12 (10–14)	61 (57–64)	34 (31–38)
**Education**								
Less than HS diploma	26 (23–29)	47 (43–51)	78 (75–82)	66 (62–70)	29 (25–32)	22 (19–25)	76 (73–79)	47 (43–52)
HS diploma	28 (25–30)	50 (46–54)	80 (77–83)	70 (67–74)	29 (26–33)	23 (20–26)	75 (72–78)	44 (41–47)
More than HS diploma	27 (24–31)	50 (45–55)	76 (72–80)	70 (66–74)	27 (23–31)	22 (18–25)	75 (71–79)	44 (40–49)
**Health insurance**								
No	36 (32–40)	55 (51–59)	80 (77–84)	71 (67–75)	29 (26–33)	24 (20–27)	79 (75–82)	52 (48–56)
Yes	26 (24–28)	48 (45–51)	78 (75–80)	67 (64–69)	27 (25–30)	21 (19–23)	71 (68–74)	41 (38–43)
Poverty level**								
At or below FPL	27 (25–29)	48 (45–51)	78 (75–80)	66 (64–69)	27 (25–30)	21 (19–23)	71 (69–74)	43 (41–46)
Above FPL	26 (23–29)	51 (46–55)	80 (76–84)	73 (68–77)	31 (27–35)	26 (22–29)	77 (72–81)	47 (43–52)
**Drug injected most frequently**								
Heroin only	27 (25–29)	49 (46–52)	77 (75–80)	66 (63–68)	25 (23–28)	20 (18–22)	71 (68–73)	39 (37–42)
Other/Multiple^††^	27 (23–30)	46 (41–50)	80 (77–84)	71 (67–75)	34 (30–37)	26 (23–29)	76 (72–79)	52 (49–56)
**Region^§§^**								
Northeast	25 (21–28)	43 (38–48)	82 (78–87)	69 (64–74)	31 (27–36)	23 (19–27)	72 (67–77)	53 (47–59)
South	26 (23–30)	50 (46–54)	78 (75–81)	68 (64–71)	25 (22–28)	19 (17–22)	73 (70–76)	42 (38–45)
Midwest	24 (20–28)	44 (39–49)	71 (66–77)	60 (55–65)	16 (13–20)	12 (10–15)	68 (62–73)	34 (30–39)
West	32 (29–36)	57 (53–61)	75 (71–78)	67 (64–71)	28 (25–32)	25 (22–28)	74 (70–77)	43 (40–47)

**Abbreviations**: CI = confidence interval; FPL = federal poverty level; HS = high school.

* Aggregate estimates are weighted averages of MSA (metropolitan statistical areas)-level percentages. MSA-level percentages were adjusted for differences in recruitment and, the size of participant persons who inject drugs peer networks, then proportionally weighted by the size of the persons who inject drugs population in each city.

^†^ Receptive syringe sharing was defined as “using needles that someone else had already injected with,” and receptive injection equipment sharing was defined as using equipment such as cookers, cottons, or water used to rinse needles or prepare drugs “that someone else had already used.” Condomless vaginal or anal sex was defined as “sex without a condom.”

^§^ Aggregate estimates for “Other” race/ethnicity excluded due to insufficient data. “Other” includes American Indian/Alaska Natives, Asians, Native Hawaiian or other Pacific Islanders, and persons of multiple races.

^¶^ Persons of Hispanic ethnicity might be of any race or combination of races.

** Poverty level is based on household income and household size.

^††^ Other drugs injected alone or two or more drugs injected with the same frequency.

^§§^ The Northeast region includes the MSAs of Boston, Massachusetts; Nassau-Suffolk, New York; New York, New York; Newark, New Jersey; and Philadelphia, Pennsylvania. South region includes Atlanta, Georgia; Baltimore, Maryland; Dallas, Texas; Houston, Texas; Miami, Florida; New Orleans, Louisiana; and Washington, District of Columbia. Midwest region includes Chicago, Illinois and Detroit, Michigan. West region includes Denver, Colorado; Los Angeles, California; San Diego, California; San Francisco, California; and Seattle, Washington. San Juan, Puerto Rico was not included.

In the 12 months preceding the interview, 58% of HIV-negative participants received an HIV test, 26% participated in an HIV behavioral intervention, 52% received syringes from syringe services programs and 34% received all their syringes from sterile sources (Table 3). Ever testing for HCV was reported by 82% of participants. Fewer white participants were tested for HIV in the preceding 12 months (51%) than were black (65%) and Hispanic (62%) participants. Fewer persons who inject drugs in the South obtained syringes from a syringe services program (36%) than did those in the Northeast (61%), Midwest (50%), and West (66%). Fewer persons who inject drugs from the South (26%) and West (28%) regions obtained syringes solely from sterile sources than did those the Northeast (44%) and Midwest (43%) regions.

**TABLE 3:** Estimated percentage* of HIV-negative participants who inject drugs (n = 9,639) who received testing and HIV prevention services, by selected characteristics — National HIV Behavioral Surveillance, 20 cities, United States, 2015

Characteristics	Tested for HIV infection in the previous 12 months, % (95% CI)	Participated in HIV behavioral interventions in the previous 12 months,^†^% (95% CI)	Ever tested for hepatitis C, % (95% CI)	Received syringes from SSP in the previous 12 months,^§^% (95% CI)	Received syringes from sterile sources only in the previous 12 months,^§^% (95% CI)
**Overall**	**58 (56–60)**	**26 (23–28)**	**82 (80–84)**	**52 (49–55)**	**34 (32–37)**
**Sex**					
Men	58 (55–60)	25 (22–27)	82 (80–84)	49 (45–52)	33 (30–35)
Women	62 (58–66)	28 (24–32)	83 (81–86)	57 (51–62)	38 (33–43)
**Race/Ethnicity** ^¶^					
Black, non-Hispanic	65 (62–69)	29 (25–33)	82 (79–85)	51 (47–56)	36 (33–40)
Hispanic**	62 (58–67)	27 (22–32)	78 (73–83)	53 (48–58)	38 (33–43)
White, non-Hispanic	51 (47–55)	23 (20–26)	84 (82–87)	54 (49–58)	28 (25–31)
**Age group (yrs)**					
18–29	58 (53–64)	23 (18–28)	74 (69–78)	46 (39–54)	26 (21–31)
30–39	58 (52–63)	20 (16–24)	84 (81–87)	54 (48–61)	30 (25–35)
40–49	61 (57–64)	31 (26–35)	82 (78–86)	56 (52–61)	35 (31–39)
≥50	61 (57–64)	27 (23–31)	86 (83–88)	54 (50–57)	38 (35–42)
**Education**					
Less than HS diploma	58 (54–62)	24 (20–28)	81 (79–84)	53 (48–57)	34 (30–38)
HS diploma	61 (59–64)	24 (21–28)	82 (79–85)	52 (48–56)	35 (32–39)
More than HS diploma	55 (51–60)	29 (25–34)	84 (81–87)	50 (45–55)	31 (26–36)
**Health insurance**					
No	47 (43–51)	15 (12–18)	70 (66–74)	36 (32–40)	23 (20–27)
Yes	61 (58–63)	28 (25–31)	85 (83–87)	55 (52–59)	37 (34–40)
**Poverty level** ^††^					
At or below FPL	59 (57–62)	26 (23–29)	83 (81–85)	52 (49–56)	35 (32–37)
Above FPL	55 (51–59)	25 (21–29)	81 (78–85)	50 (44–56)	33 (28–38)
**Drug injected most frequently**					
Heroin only	58 (55–61)	26 (23–28)	83 (81–85)	54 (50–57)	36 (33–39)
Other/Multiple^§§^	59 (56–62)	26 (22–29)	80 (77–83)	45 (42–49)	29 (25–33)
**Region** ^¶¶^					
Northeast	63 (58–68)	33 (28–38)	87 (84–91)	61 (54–67)	44 (39–50)
South	62 (59–66)	23 (19–26)	79 (76–82)	36 (32–39)	26 (23–30)
Midwest	47 (42–52)	19 (15–22)	78 (74–82)	50 (44–55)	43 (37–48)
West	49 (46–53)	20 (17–23)	80 (76–83)	66 (62–70)	28 (24–31)

**Abbreviations**: CI = confidence interval; FPL = federal poverty level; HS = high school; SSP = syringe services program.

* Aggregate estimates are weighted averages of MSA (metropolitan statistical areas)-level percentages. MSA-level percentages were adjusted for differences in recruitment and the size of participant persons who inject drugs, peer networks then proportionally weighted by the size of the persons who inject drugs population in each city.

^†^ Participating in an individual or group HIV behavioral intervention (e.g., a one-on-one conversation with a counselor or an organized discussion regarding HIV prevention) did not include counseling received as part of an HIV test or conversations with friends.

^§^ Receiving a syringe from a syringe services program (SSP) was defined as reporting receiving a sterile syringe or needles at least once from an SSP or syringe/needle exchange program. Receiving syringes from sterile sources only included reporting receiving syringes from at least one of the following: SSP, pharmacy, or healthcare provider and not any other sources during the previous 12 months.

^¶^ Aggregate estimates for “Other” race/ethnicity excluded due to insufficient data. “Other” includes American Indian/Alaska Natives, Asians, Native Hawaiian or other Pacific Islanders, and persons of multiple races.

** Persons of Hispanic ethnicity might be of any race or combination of races.

^††^ Poverty level is based on household income and household size.

^§§^ Other drugs injected alone or two or more drugs injected with the same frequency.

^¶¶^ The Northeast region includes the MSAs of Boston, Massachusetts; Nassau-Suffolk, New York; New York, New York; Newark, New Jersey; and Philadelphia, Pennsylvania. South region includes Atlanta, Georgia; Baltimore, Maryland; Dallas, Texas; Houston, Texas; Miami, Florida; New Orleans, Louisiana; and Washington, District of Columbia. Midwest region includes Chicago, Illinois and Detroit, Michigan. West region includes Denver, Colorado; Los Angeles, California; San Diego, California; San Francisco, California; and Seattle, Washington. San Juan, Puerto Rico was not included.

Among persons who inject drugs, a higher percentage of those with health insurance were tested for HIV infection in the previous 12 months (61%) than were those without health insurance (47%) (Table 3). Similarly, more persons who inject drugs with health insurance reported participating in HIV behavioral interventions (28%) or ever testing for HCV infection (85%) than did those without health insurance (15% and 70%, respectively).

## Discussion

This report provides updated prevalence of HIV infection and behaviors since the last NHBS survey among persons who inject drugs in 2012 (*3*). In 2015, persons who inject drugs continued to report high levels of injection and sex risk behaviors placing them at increased risk for HIV acquisition, highlighting the need for effective and comprehensive prevention services, including access to sterile injection equipment.

The prevalence of HIV infection was 7% (CI = 6%–8%) in 2015, lower than in 2012 (11%; 95% CI = 9%–12%). The change might partially be explained by the differences in the sample composition from 2012 to 2015: the percentage of white persons who inject drugs increased from 30% in 2012 to 39% in 2015, and white persons who inject drugs in 2012 and 2015 had the lowest HIV prevalence (5% and 6%, respectively).

Consistent with previous reports (*3*), this analysis found a higher prevalence of HIV infection among blacks who inject drugs than among whites who inject drugs, despite fewer reported risk behaviors among blacks. In 2015, when compared with white persons who inject drugs, fewer black persons who inject drugs shared syringes or injection equipment, fewer had condomless vaginal or anal sex, more tested for HIV infection, and more received syringes only from sterile sources in the previous 12 months. Taken together with data from previous reports suggesting that persons who first injected drugs during the 5 years before their interview and young persons who inject drugs are more likely to be white (*2*), these findings suggest HIV prevalence among white persons who inject drugs could be lower because they have had less time to acquire HIV infection through injection drug use.

Overall, higher percentages of 2015 participants tested for HIV infection in the previous 12 months (51% in 2012, 58% in 2015) and ever tested for HCV (78% in 2012, 82% in 2015) (*3*). Increases in HIV and HCV testing could be the result of increased access to health insurance among persons who inject drugs (69% in 2012, 82% in 2015) (*3*). In 2015, higher percentages of persons who inject drugs and who have health insurance tested for HIV infection, participated in HIV behavioral interventions, and ever tested for HCV than did those without health insurance. Although these results highlight gains in HIV and HCV testing measures, nearly half of persons who inject drugs did not test for HIV in the previous 12 months as recommended by CDC (*8*). Continued activities that expand HIV testing in settings that provide services to persons who inject drugs, such as in syringe services programs, substance use disorder treatment programs and emergency departments, are needed.

The findings in this report are subject to at least four limitations. First, because a method of obtaining standard probability samples of persons who inject drugs does not exist, the representativeness of the NHBS sample cannot be determined. Although adjusted for RDS (*5*), biases related to participants’ recruitment behavior or their willingness and ability to participate in the interview might have affected the sample. Second, the numbers of participants in some cities were insufficient to include these cities in the aggregate estimates. The number of cities excluded from aggregate estimates varied based on the analysis variable. Third, persons who inject drugs were interviewed in 20 cities with high prevalences of HIV infection; findings from these cities might not be generalizable to all persons who inject drugs including those who reside in rural or nonmetropolitan areas. Finally, behavioral data are self-reported and subject to social desirability bias.

This analysis highlights the ongoing need for risk reduction and HIV prevention services among persons who inject drugs. Only half of persons who inject drugs used syringe services programs and only a third obtained their syringes exclusively from sterile sources. Access to sterile injection and drug preparation equipment is critical for the prevention of HIV infections among persons who inject drugs. Although access to syringes through syringe services programs has increased in the United States (*9*), the available supply is likely insufficient to meet the demand, and multiple areas continue to lack access to these services. The recent opioid use epidemic increases the potential for HIV outbreaks among persons who inject drugs, particularly in areas with limited prevention services for persons who inject drugs (*4*). Thus, failure to respond appropriately to this prevention gap could reverse earlier successes in reducing HIV infection among persons who inject drugs (*2*). Comprehensive syringe services programs reduce transmission of HIV and other infections (*10*) by providing access to safe syringe disposal; risk reduction education; HIV and viral hepatitis testing; referrals to health services including treatment for HIV, HCV, or substance use disorder (including medication-assisted therapy) and mental health disorders; and preexposure prophylaxis. Recent changes in federal appropriations law^††††^ permitting the use of federal funding to support syringe services programs present an opportunity to improve access to these critical prevention services to persons who inject drugs.

**Summary**

**What is already known about this topic?**

Persons who inject drugs are at increased risk for acquiring human immunodeficiency virus (HIV) infection. In 2012, National HIV Behavioral Surveillance found an overall 11% prevalence of HIV infection of among persons who inject drugs living in 20 large cities. Among HIV-negative persons who inject drugs, 27% shared syringes and 67% had vaginal sex without a condom in the previous 12 months.

**What is added by this report?**

In 2015, National HIV Behavioral Surveillance found a 7% prevalence of HIV infection among persons who inject drugs which was lower than in 2012 (11%). Among HIV-negative respondents, 27% reported sharing syringes and 67% reported having vaginal sex without a condom in the previous 12 months; only 52% received syringes from a syringe services program and 34% received all syringes from sterile sources. HIV infection prevalence was higher among blacks (11%) than whites (6%) but more white persons who inject drugs shared syringes (white: 39%; black: 17%) and injection equipment (white: 61%; black: 41%) in the previous 12 months**.**

**What are the implications for public health practice?**

Persons who inject drugs are at risk for acquiring HIV infection because of their drug use practices and sexual behaviors. Approximately half of injection drug users did not receive syringes from a syringe services program in the previous 12 months. Provision of sterile syringes and other community-based strategies can decrease risk for HIV transmission. Persons who inject drugs need access to sterile injection and drug preparation equipment; HIV and viral hepatitis testing; health services that provide treatment for HIV infection, viral hepatitis, substance use disorder and mental health disorders; preexposure prophylaxis; and education on drug- and sex-related risks and risk reduction.

## Footnotes

* The 20 metropolitan statistical areas (MSAs) were Atlanta, Georgia; Baltimore, Maryland; Boston, Massachusetts; Chicago, Illinois; Dallas, Texas; Denver, Colorado; Detroit, Michigan; Houston, Texas; Los Angeles, California; Miami, Florida; Nassau-Suffolk, New York; New Orleans, Louisiana; New York, New York; Newark, New Jersey; Philadelphia, Pennsylvania; San Diego, California; San Francisco, California; San Juan, Puerto Rico; Seattle, Washington; District of Columbia.

† Recruitment chains in each city began with three to 15 initial participants identified during formative assessment. Initial participants who completed the interview were asked to recruit up to five other persons who inject drugs using a coded coupon system designed to track referrals. Referred and surveyed persons who inject drugs were also asked to recruit up to five other persons who inject drugs.

§ Persons who injected drugs during the previous 12 months, resided in the MSA, were aged ≥18 years and could complete the interview in English or Spanish.

¶ All 20 MSAs conducted HIV screening with a rapid test; for supplemental testing to confirm rapid tests, four collected blood via venipuncture, 15 collected blood via dried blood spots, and one collected oral samples.

** The incentive format (cash or gift card) and amount varied by MSA based on formative assessment and local policy. A typical format included $25 for completing the interview, $25 for providing a specimen for HIV testing, and $10 for each successful recruitment (maximum of five).

†† Receptive sharing of syringes was defined as “using needles that someone else had already injected with,” and receptive sharing of injection equipment was defined as using equipment such as cookers, cottons, or water used to rinse needles or prepare drugs “that someone else had already used.”

§§ Condomless vaginal sex/condomless anal sex was defined as sex without a condom at least once in the past 12 months. Ascertainment of male-to-male anal sexual contact was restricted to males and includes both insertive and receptive anal sexual contact.

¶¶ Participating in an individual or group HIV behavioral intervention was defined as a one-on-one conversation with a counselor or an organized discussion regarding prevention of HIV infection, and did not include counseling received as part of an HIV test or conversations with friends.

*** Receiving a syringe from a syringe services program was defined as receiving a sterile syringe or a needle at least once from a “needle or syringe exchange program” during the previous 12 months. Receiving syringes from sterile sources only was defined as receiving all syringes from syringe services program, pharmacy, or health care provider and not any other sources during the previous 12 months.

††† Behavioral analyses from previous reports excluded participants reporting a previous HIV-positive test result. A comparison of analysis excluding those who tested HIV-positive to analysis excluding those who reported a previous HIV-positive test did not yield significantly different estimates.

§§§ For city-level estimates for which confidence intervals could not be calculated, maximally wide confidence intervals (0–1) were used in aggregation. City-level estimates with insufficient data for analysis were excluded from the aggregated estimates. Estimates not including all cities represented 16% of the analysis.

¶¶¶ Data from 331 participants were excluded because of missing recruitment data, lost data during electronic upload, incomplete survey data, survey responses with questionable validity, invalid HIV test results, or the participant could not be identified as male or female. Reasons for exclusion were not mutually exclusive.

**** In comparing HIV prevalence among black persons who inject drugs to white persons who inject drugs, the confidence intervals (CIs) appear to overlap. However, this is because of rounding as the actual CIs do not overlap (black persons who inject drugs: 11.5% [95% CI = 8.4–14.6]; white persons who inject drugs: 6.4% [95% CI = 4.7–8.2]).

†††† Consolidated Appropriations Act, 2016. Division H, Section 520. https://www.congress.gov/114/bills/hr2029/BILLS-114hr2029enr.pdf.
